# WHO GETS HELP? A NATIONAL LONGITUDINAL STUDY OF PERSONAL NETWORKS AND PANDEMIC SUPPORT AMONG OLDER ADULTS

**DOI:** 10.1093/geroni/igac059.120

**Published:** 2022-12-20

**Authors:** Molly Copeland, Hui Liu

**Affiliations:** Michigan State University, East Lansing, Michigan, United States; Michigan State University, East Lansing, Michigan, United States

## Abstract

Personal networks are a key component in the provision of social support for older adults. Such support is particularly critical during the COVID-19 pandemic, when traditional avenues of social engagement or assistance are disrupted. Here, we use nationally representative data from the National Social Life, Health, and Aging Project that assesses older adults’ pre-pandemic personal networks and receiving instrumental help and emotional support during the pandemic. We find that larger pre-pandemic confidant networks predict higher odds of receiving needed help and support, higher odds of receiving help and support more often than before the pandemic, and lower odds of being unable to get help. Denser pre-pandemic networks also predict higher odds of receiving instrumental help and support during the pandemic, while having a greater proportion of kin in pre-pandemic networks predicts higher odds of receiving pandemic help for non-white older adults only. Together, results suggest that features of older adults’ pre-pandemic confidant network structure and composition can provide underlying conditions for receiving social support during the pandemic.

